# FKBP51 and FKBP12.6—Novel and tight interactors of Glomulin

**DOI:** 10.1371/journal.pone.0221926

**Published:** 2019-09-06

**Authors:** Andreas Hähle, Thomas M. Geiger, Stephanie Merz, Christian Meyners, Mao Tianqi, Jürgen Kolos, Felix Hausch

**Affiliations:** Institute for Organic Chemistry and Biochemistry, Technische Universität Darmstadt, Darmstadt, Germany; Université de Genève, SWITZERLAND

## Abstract

The protein factor Glomulin (Glmn) is a regulator of the SCF (Skp1-CUL1-F-box protein) E3 ubiquitin-protein ligase complex. Mutations of Glmn lead to glomuvenous malformations. Glmn has been reported to be associated with FK506-binding proteins (FKBP). Here we present *in vitro* binding analyses of the FKBP—Glmn interaction. Interestingly, the previously described interaction of Glmn and FKBP12 was found to be comparatively weak. Instead, the closely related FKBP12.6 and FKBP51 emerged as novel binding partners. We show different binding affinities of full length and truncated FKBP51 and FKBP52 mutants. Using FKBP51 as a model system, we show that two amino acids lining the FK506-binding site are essential for binding Glmn and that the FKBP51-Glmn interaction is blocked by FKBP ligands. This data suggest FKBP inhibition as a pharmacological approach to regulate Glmn and Glmn-controlled processes.

## Introduction

**Fig 1:**
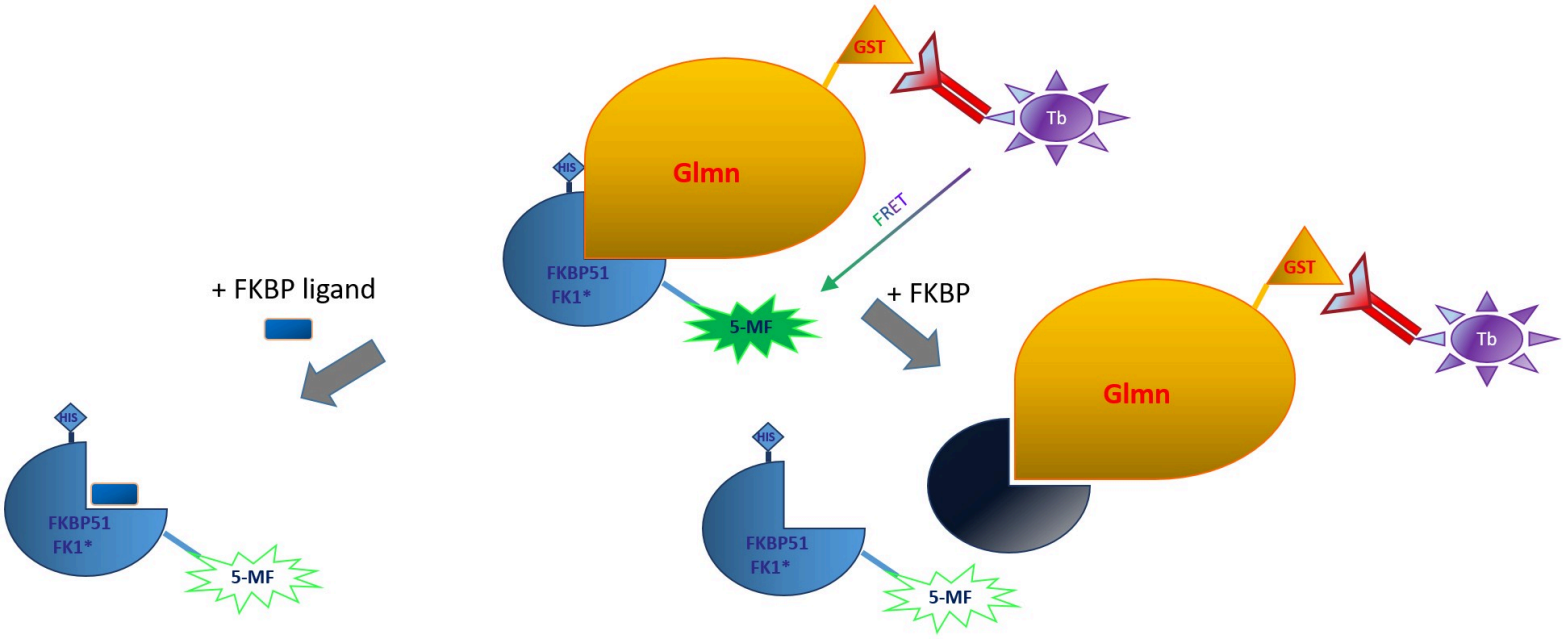
HTRF assay setup. Characterization of the binding of Glmn and the FK1 domain of FKBP51* (MonoCys mutant covalently labelled with Fluorescein-5-Maleimide = 5-MF) via a homogeneous time-resolved fluorescence resonance energy transfer (HTRF) assay. Molecules that bind to the binding sites on FKBPs or Glmn can be quantified in a competitive setup. HIS = polyhistidine-tag, GST = glutathione S-transferase tag.

Glmn regulates the Skp1-Cullin-F-box complex, an E3-Ligase, which primes proteins for proteasomal degradation [1]. One of the most studied examples of this E3 ligase family is CRL1^Fbw7^ [2]. Glmn binds to this complex by intercalating between Cul1 and Rbx1. Henceforth, Glmn masks the interaction surface of Rbx1 towards the E2 ubiquitin-conjugating enzyme Cdc34, leading to an inhibition of the ligase activity [3]. Glmn is thought to be a regulator of ligase function and to prevent an overshooting ubiquitinylation reaction, which is mediated by Rbx1-binding cullins. This was further supported by a crystal structure of the Glmn-Cullin complex [3]. Later, an interaction of Glmn with cellular inhibitor of apoptosis proteins 1 and 2 (cIAP1 and cIAP2), which regulate E3 ligases, was discovered [4]. Mutations of the Glmn gene are the main cause of glomuvenous malformations [5–7] although the underlying mechanism remains unclear and the link to E3 ligase regulation remains to be elucidated. Additionally, most recently Glmn was associated with the infectious mechanism of Shigella [4,8], where Glmn was claimed to be hijacked by a bacterial E3 ligase to promote inflammation. Glomulin was initially described as FKBP Associated Protein (called FAP48 or FAP68) [5,9]. In these studies, the FK506-binding proteins FKBP12 and 52 were identified as interaction partners of Glmn in a yeast-2-hybrid system [9,10]. Furthermore, Pro219 of Glmn was suggested to be essential for the interaction with FKBP12 and FKBP52 [10]. More recently, a link between FKBP51 and Glmn was described in a high-throughput interactome network study [11]. During the last decade, FKBP51 emerged as a player in various pathologies. As a key regulator of the hypothalamus-pituitary-adrenal axis, FKBP51 knockout leads to an improved stress regulation in mice. Furthermore, FKBP51 knockout mice exhibit resistance to diet-induced weight gain as well as experimentally induced chronic pain [12]. Selective ligands, which discriminate FKBP51 over its close homologue FKBP52, were shown to positively influence stress-coping behavior, reduce weight gain and to ameliorate hypersensitivity states of chronic pain. In neuronal cells, overexpression of FKBP51 retards neurite elongation during neuronal differentiation, while knockdown and pharmacological inhibition increases it [13]. FKBP52 was shown to be essential in mice for the correct development of reproductive organs, likely via its action on sex hormone receptors [14]. FKBP12 and FKBP12.6 bind to ryanodine receptors and to receptors of the TGFβ family, where they are believed to suppress leaky signalling [13]. To elucidate a potential regulation of Glmn by FKBPs and to increase our understanding of their molecular action modes we determined the binding affinities of Glmn towards various FKBPs and the details of this interaction (Fig 1).

## Methods

### Overview of proteins

Genes encoding codon optimized His-FKBP51FK1-MonoCys (aa1-140, C103A, C107I, E140C) and His-FKBP12-MonoCys (C23V / E108C) were obtained from GeneArt^®^ (ThermoFisher Scientific) and subcloned into pET30b expression vector. The mutated cysteines are neither located within the binding site nor conserved between FKBP51, FKBP52 and FKBP12. The corresponding internal cysteines were mutated in order to ensure labelling at only a single, C-terminal site by exchanging Glu140 in FKBP51FK1 and Glu108 in FKBP12, respectively.

His-FKBP51FK1 (aa16-140) in pET30b expression vector; His-FKBP51FK1, His-FKBP51FK1 (F67V), His-FKBP51FK1 (FD67/68DV), His-FKBP52FK1, His-FKBP51-Strep, His-FKBP52-Strep, His-FKBP12 and His-FKBP12.6 in pProEx-HTA and GST-Glmn in pGex expression vector were used as previously described [3,15–19].

### Protein purification

All used proteins were expressed in *E*.*coli* BL21(DE3) cells. The cultures were grown to an OD_600_ = 0.5, induced with 600 μM IPTG, and grown for 4 h at 37 °C (Mono-Cys variants for 4 h at 30 °C and GST-Glmn at 18 °C overnight). Cultures were spun down, resuspended in lysis buffer (20 mM HEPES, 200 mM NaCl, 200 mg/mL lysozyme, 2.5 mM PMSF, 0.1 mg/mL DNAse I, pH 8.0) and subjected to sonication. After that, the lysate was centrifuged (35k xg, 4 °C, 30 min). Nickel-NTA beads (Machery Nagel) or Glutathion beads (GE Healthcare) were equilibrated in washing buffer (20 mM HEPES, 200 mM NaCl, pH 8.0), added to the supernatant and incubated for 2 h on a rolling device at 4 °C. Afterwards, the beads were spun down (100 xg, 2 min), the supernatants were removed and the beads were washed two times in washing buffer. The beads were transferred to a column (Bio-Rad) and washed again. Elution was performed with elution buffer (20 mM HEPES, 200 mM NaCl, pH 8.0, 300 mM imidazole or 10 mM glutathione, respectively). The elution progress was monitored via a qualitative Bradford assay. Protein-containing fractions were pooled, centrifuged (14k xg, 4 °C, 20 min) and the supernatant was subjected to size exclusion chromatography (FPLC buffer: 20 mM Hepes, 20 mM NaCl, 5%(v/v) glycerol, pH 8.0). Resulting protein fractions were quantified via molecular extinction at 280 nm, frozen in liquid nitrogen and stored at -80 °C. All buffers used for the purification of Mono-Cys mutants and GST-Glmn additionally contained 5 mM DTT.

### Protein labelling

FKBP51FK1-MonoCys and FKBP12-MonoCys were dialysed for 5 cycles in a Dialyse Slide-A-Lyzer 3.5k MWCO (ThermoFisher) in 20 mM HEPES, 20 mM NaCl, 5%(v/v) glycerol, 10 mM TCEP pH 8.0 to remove DTT. The protein was added to equilibrated Ni-NTA. After 2 h, a 20-fold molar excess of fluorescein-maleimide (Toronto Research Chemical) was added and incubated for 2 h. The mix was added to a column (Bio-Rad) and washed with buffer (20 mM HEPES, 20 mM NaCl, 5% (v/v) glycerol, pH 8.0, 5 mM DTT) until the flowthrough became colorless. Elution was performed with elution buffer (20 mM HEPES, 200 mM NaCl, pH 8.0, 300 mM imidazole). The elution was stopped as the eluate became colorless. In order to remove remaining unreacted fluorescein-maleimide, the labelled protein was dialysed for 5 cycles in a Dialyse Slide-A-Lyzer 3.5k MWCO (ThermoFisher) in 20 mM HEPES, 20 mM NaCl, 5% glycerol, 5 mM DTT, pH 8.0. Protein concentration was determined by measuring OD_280_ and OD_495_ (calculation protocol by ThermoFisher).

### Labelled protein activity assay (destructive FRET)

The TAMRA-labelled bicyclic ligand FK[4.3.1]-16g [20] (S1A Fig) was serially diluted in 15 steps to a concentration series ranging from 25 μM to 1.5 nM in DMSO. 1 μL of each dilution step was added to a black 384 well plate (Corning 3575). 50 μL of a 5 nM solution of Fluorescein-5-Maleimide (5-MF)-labelled protein in FP-Assay buffer (20 mM HEPES, 0.002%(v/v) Triton X-100, pH 8.0) were added to each well. Fluorescence (Ex: 485 nm, Em: 520 nm) was measured with a Tecan Genios Pro plate reader. Experiments were performed in triplicates. Mean values and standard deviations were plotted. Curves were fitted via a one-site-ligand depletion curve (Y = A / E * 0.5 *(X + E + 1 / K—sqrt (sqrt (X + E + 1 / K)—4 * E * X))+B) using GraphPad Prism 6. Figure tables show K values and their respective standard deviation.

### Active site titration of FKBPs

All unlabelled FKBPs were quantified by active site titrations [15]. Briefly, proteins were serially diluted in 15 steps in FP-assay buffer and mixed 1:1 with a 50 nM solution of the TAMRA-labelled bicyclic ligand FK[4.3.1]-16g [20] (S1A Fig) in FP-Assay buffer in a black 384 well plate (Corning 3575). Fluorescence polarization was measured with a Tecan Genios Pro plate reader, plotted against the UV concentration and subjected to a four-parameter fit using GraphPad Prism6: Y = Bottom + (Top—Bottom) / (1 + (x/EC50)^(-Hillslope)). Experiments were performed in either duplicates or triplicates. Mean values and standard deviations were plotted. Concentration of active protein was calculated: *c*_*AST*_ *= ((0*.*5x c*_*Tracer*_ *+ K*_*D*_*) / EC*_*50*_*) x c*_*UV*_. The results of the active site titrations were used for the HTRF assays and can be found in S1 Table.

### HTRF-assay

Equal parts (20 μL) of 120 nM FKBP51FK-MonoCys-Fluorescein (FKBP51FK1-5-MF), competitor solution (unlabelled FKBPs or FKBP ligands at various concentrations), 120 nM GST-Glmn and 3.2 nM MAb Anti GST-Tb cryptate (61GSTTLA, Cisbio) in HTRF buffer (20 mM HEPES, 5%(v/v) glycerol, 20 mM NaCl, 10 mM DTT, pH 8.0) were consecutively added to a black 384 well plate (Corning 3575) and incubated at room temperature for 1h. Fluorescence (Ex: 485 nm, Em: 520 / 620 nm) was measured with a Tecan Genios Pro plate reader in HTRF mode (lag time: 150 μs, integration time: 500 μs). The signal of 520 nm was normalized to the signal of 620 nm as a reference. Experiments were performed in triplicates. Mean values and standard deviations were plotted. Curves were fitted via a four-parameter fit: Y = Bottom + (Top-Bottom)/(1+10^((LogIC50-X)*HillSlope)) using GraphPad Prism 6. Figure tables show IC_50_ values and their respective standard deviation.

## Results

**Fig 2:**
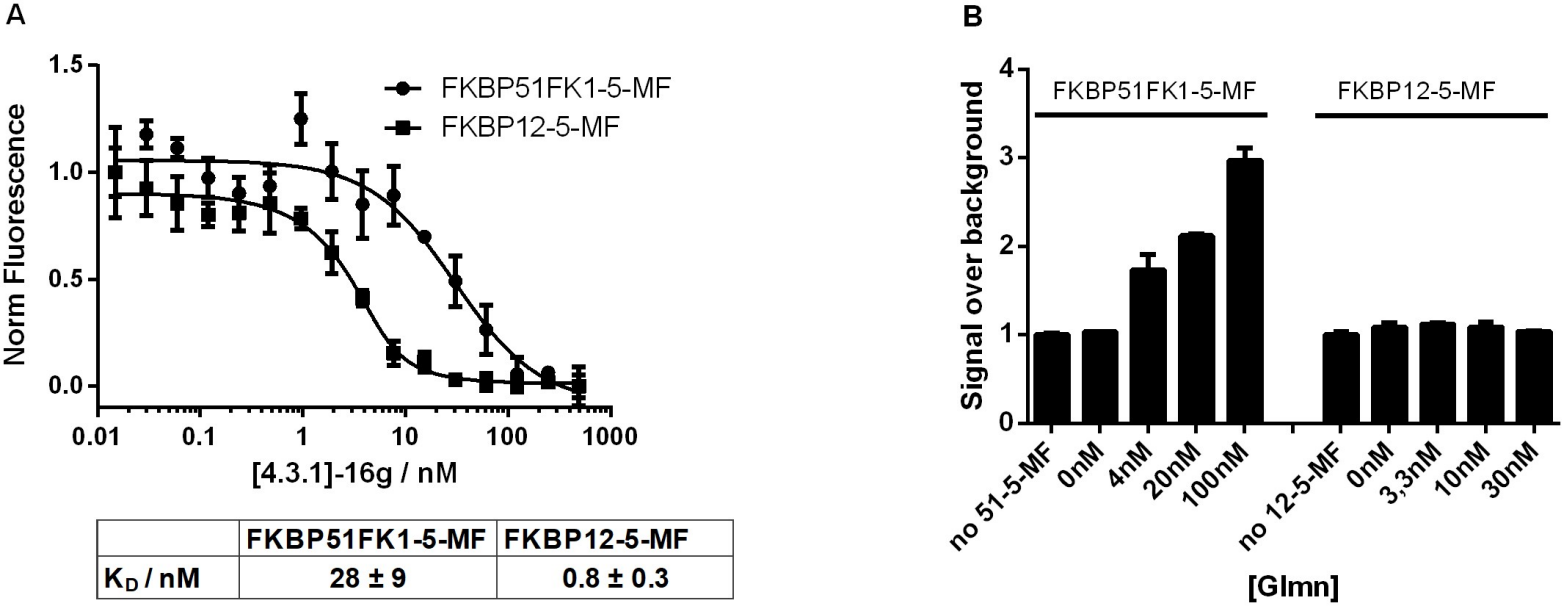
Development of an HTRF-based assay for binding of FKBPs and Glmn. (A) Fluorescein-labelled FKBP51FK1 and FKBP12 bind to the TAMRA-labelled FKBP ligand FK[4.3.1]-16g as observed by a destructive FRET. Corresponding K_D_ values are indicated below and represent mean values and standard deviation of three independent dilution series; (B) Titration of GST-Glmn to 30nM Fluorescein-labelled protein in the presence of 0.8 nM Anti GST-Tb cryptate followed by HTRF readout (Ex: 485 nm, Em: 520 / 620 nm).

To quantify the Glmn-FKBP interactions we developed a protein-protein interaction assay with purified proteins. Towards this goal, we chose FKBP12 and the FK506-binding domain of FKBP51 as a well described and structurally very well understood representatives of the FKBP family. We generated labelled mutants of FKBP51FK1 and FKBP12 containing a single cysteine at their respective *C*-terminus with Fluorescein-5-Maleimide. In order to confirm the activity of the labelled proteins, a destructive FRET assay was performed using a TAMRA-labelled FKBP ligand as FRET acceptor [20]. The fluorescein label emission of both proteins was dose-dependently quenched upon the titration with the FRET acceptor (Fig 2A). Importantly, the apparent affinities were very similar compared to the affinities, which were obtained for this tracer for the wild type protein constructs [20], suggesting that the labelled proteins were fully binding-competent and that the mutations of the internal cysteines did not compromise the principal binding capacity of the FK506-binding site.

To set up a direct FKBP-Glmn protein-protein binding assay, both labelled FKBPs were incubated with increasing amounts of GST-tagged Glmn and a Terbium-cryptate-linked antibody against this tag. Surprisingly, an HTRF signal was only obtained with the FK1 domain of FKBP51 but not with FKBP12, when using the same batch of protein preparation as used for the destructive FRET assay (Fig 2B).

Based on these results, 30 nM FKBP51FK1-5-MF and 30 nM GST-Glmn were chosen to set up a competitive HTRF assay.

**Fig 3:**
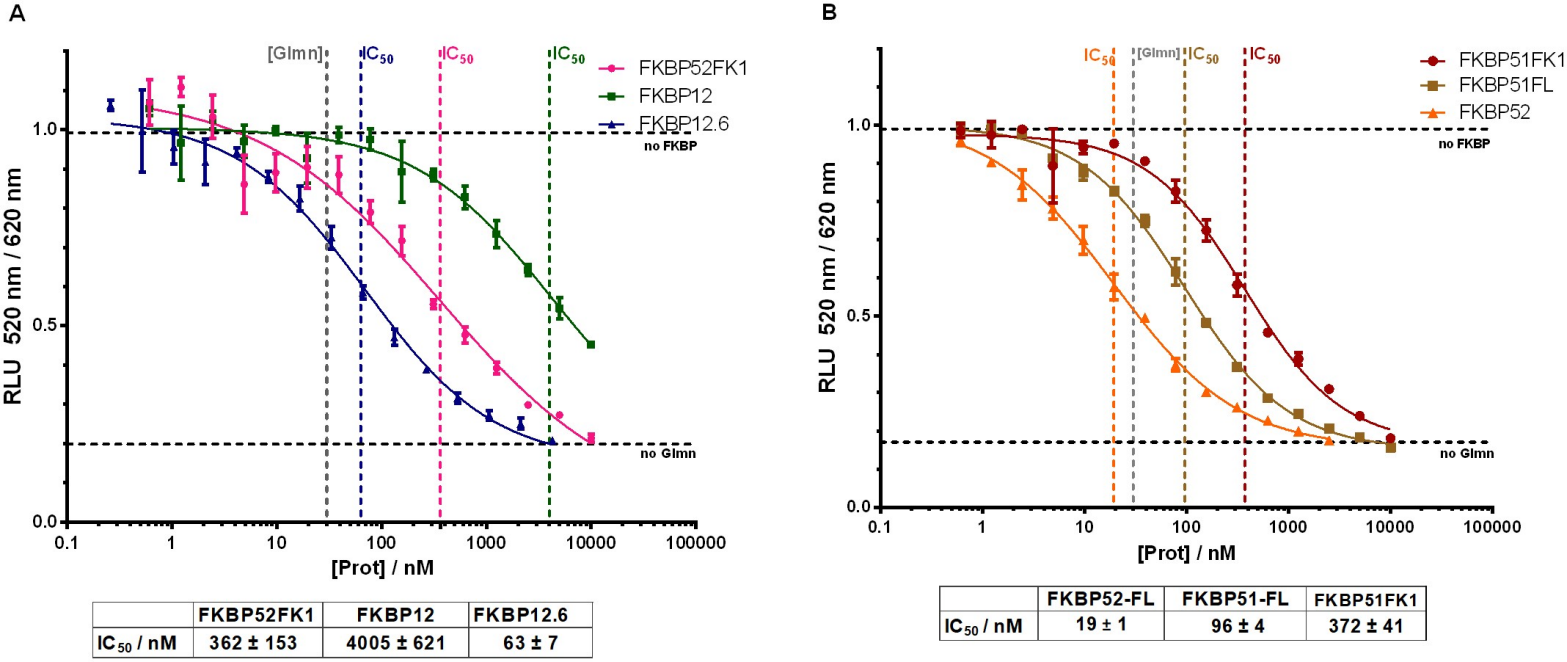
Different unlabelled FK1 domains and FKBPs compete for Glmn binding. (A), FKBP52FK1, FKBP12 and FKBP12.6; (B) FKBP52FL (full length), FKBP51FL (full length), FKBP51FK1 (aa 16–140). Corresponding IC_50_ values are indicated below and represent mean values and standard deviation of three independent dilution series.

In the literature, Glmn has been described as an interactor of FKBP52 and FKBP12 [9,10]. Using the competitive HTRF setup, we titrated various unlabelled FKBPs (Fig 3). Prior to these experiments, all used FKBPs were characterized via active site titration or isothermal titration calorimetry to confirm the concentration of binding-competent proteins after purification (shown in S2 and S3 Figs)[15,20].

We observed that the FK1 domains of FKBP51 and FKBP52 as well as FKBP12.6 competed for Glmn with comparable potency (Fig 3). Notably, full-length FKBP52 competed for Glmn binding approximately 19-fold better than its FK1 domain, indicating additional binding contacts exhibited by its other domains. The difference between the isolated FK506-binding domain and full-length protein was much weaker for FKBP51 (4-fold), indicating that the FK2 and TPR domain of FKBP51 also contact Glmn but that these interactions are weaker compared to FKBP52.

Even more interesting was the observed difference between FKBP12 and FKBP12.6, which both comprise a single, highly homologous FK506 binding domain. While FKBP12.6 binds Glmn with similar affinity as the full-length FKBP51, FKBP12 competed for Glmn-binding only very weakly. However, the poor competition of FKBP12 is in line with the inactivity of the labelled FKBP12 in the direct FRET assay (Fig 2B).

**Fig 4:**
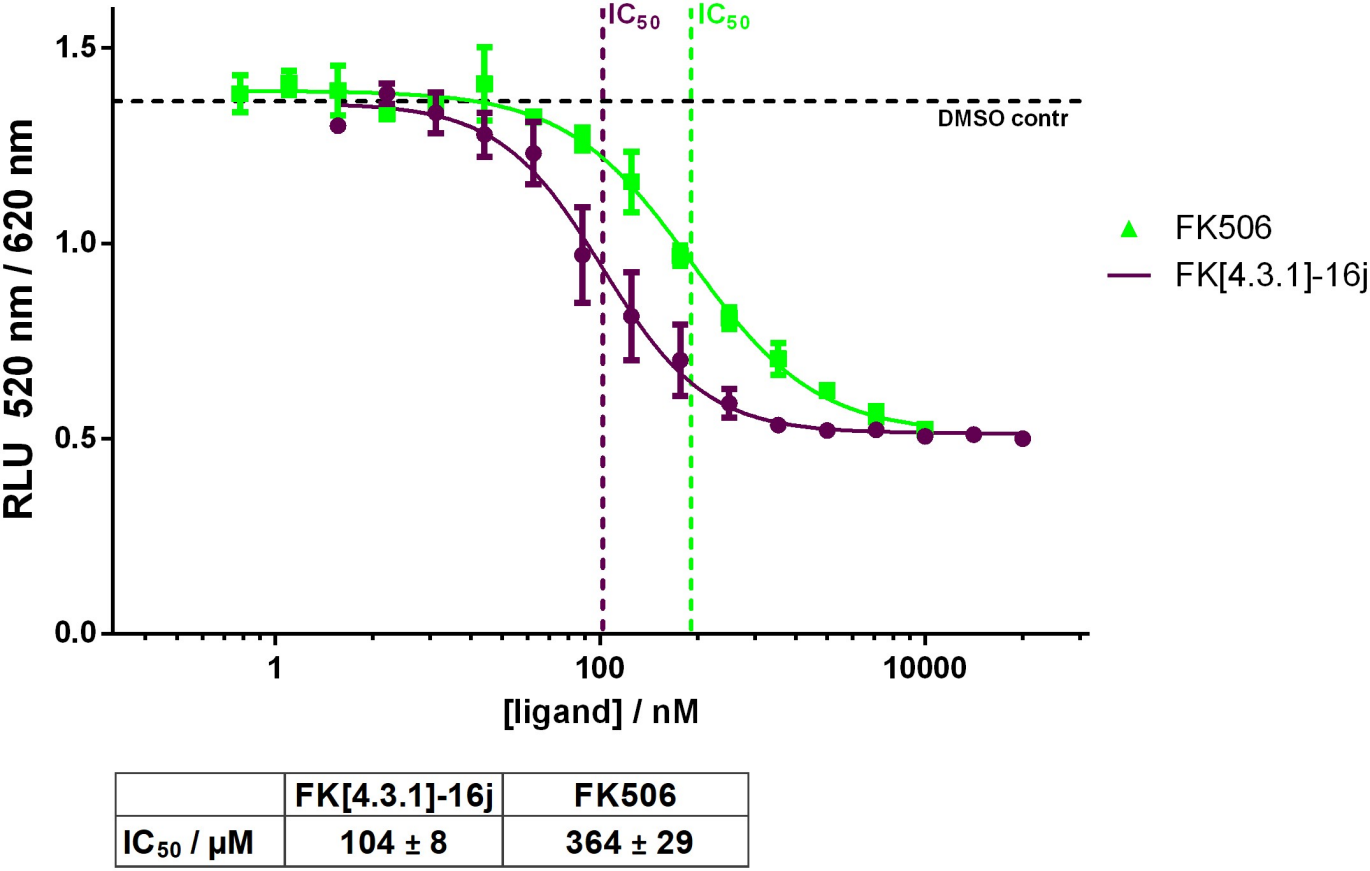
The unlabelled FKBP ligands FK506 and FK[4.3.1]-16j compete for Glmn binding in the HTRF assay. Corresponding IC_50_ values are indicated below and represent mean values and standard deviation of three independent dilution series.

To further probe the FKBP51-Glmn interaction, we tested a competition of small molecule FKBP ligands in the competitive HTRF assay (Fig 4). The natural ligand FK506 as well as the potent bicyclic analog [4.3.1]-16j [20] efficiently blocked the FKBP51-Glmn interaction, supporting the notion that the FK506-binding pocket is likely the major Glmn binding site in FKBP51. However, at this point, we cannot exclude that the compounds FK506 and FK[4.3.1]-16j block the Glmn-FKBP interaction through moieties extruding from the binding pocket, which might sterically interfere with Glmn binding.

**Fig 5:**
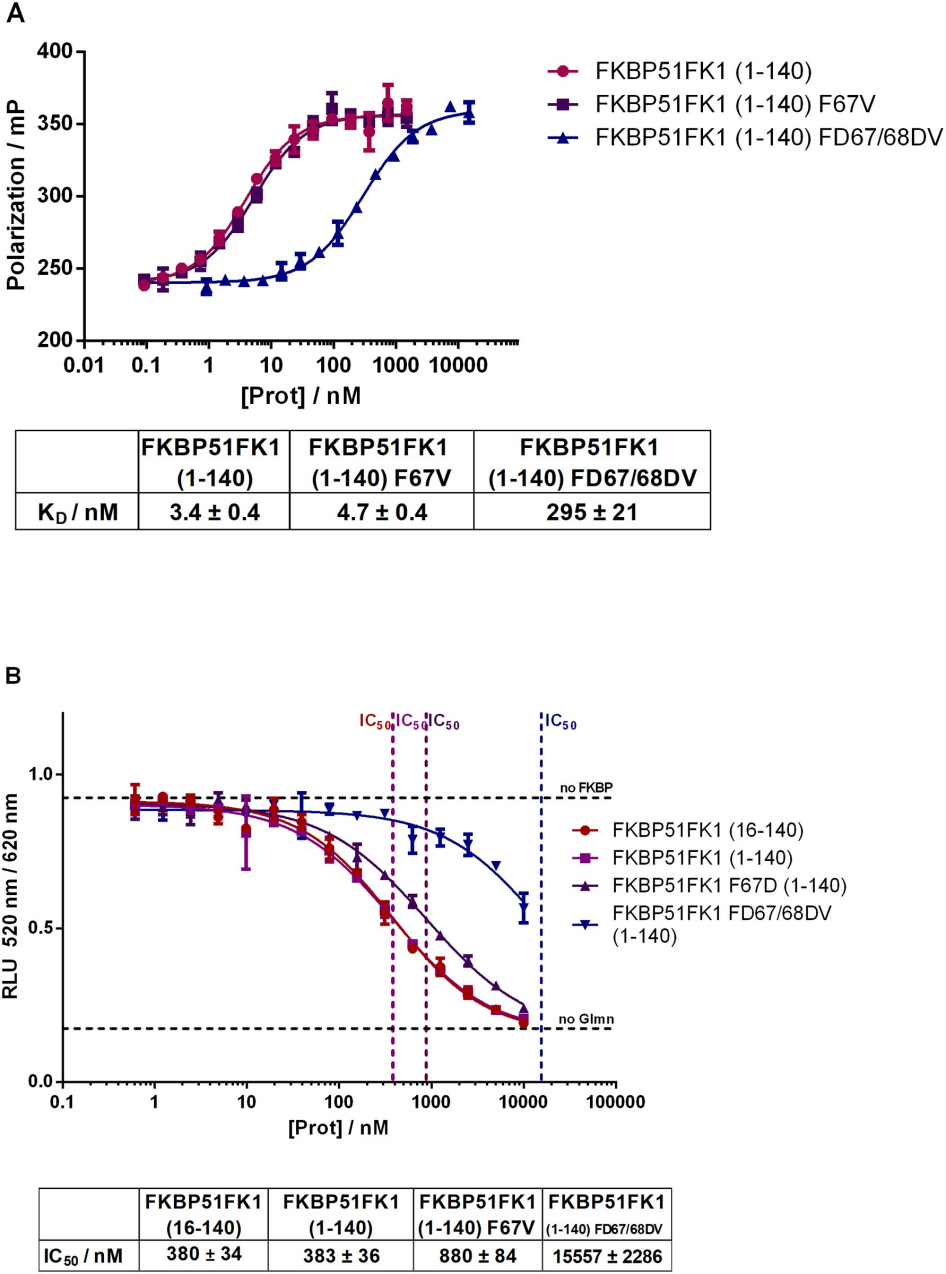
Characterization of FKBP51 active site mutants. (A) FKBP51FK1 (1–140) and mutants F67V bind to the high-affinity TAMRA-labelled tracer FK[4.3.1]-16g with similar affinity in the fluorescence binding assay, while binding of the mutant FD67/68DF is compromised. (B) FKBP51 mutants F67V bind with slightly and FD67/68DV with substantially reduced affinity to GST-Glmn in the competitive HTRF assay. K_D_ or IC_50_ values are indicated below and represent mean values and standard deviation of three independent dilution series.

To confirm the FK506-binding site in FKBPs as the major Glmn binding pocket, we investigated known FK506-binding site mutations using FKBP51 as a model system. Specifically, we tested the FKBP51FK1 mutants carrying an F67V [21,22] or an FD67/68DV [23] double mutation, which line the active site of the FK506-binding pocket. The F67V mutation is known to retain anti-neuritotrophic activity as well as binding to bicyclic FK506 analogs [24], while the FD67/68DV double mutation was shown to abolish the peptidyl-prolyl isomerase activity of FKBP51 [23,25–27]. The affinity of the fluorescent tracer TAMRA-labelled bicyclic ligand FK[4.3.1]-16g was only minimally shifted by the F67V mutation, while the binding capacity of the FD67/68DV double mutation was substantially compromised (Fig 5A). The integrity of all FKBP51 mutant batches was confirmed by active site titration by fluorescence polarization or isothermal calorimetry (S3 Fig).

In the competitive HTRF assay, the F67V mutant exhibited slightly reduced affinity to Glmn, whereas the competition for the FD67/68DV double mutant was very weak (Fig 5B). This indicates that especially D68 seems to be important for the binding of Glmn to FKBP51. Interestingly, in this assay we also tested complete FK1(1-140aa) in direct comparison to the previously used *N*-terminally truncated FK1(16-140aa) construct [18] and observed that the presence of the *N*-terminus did not reduce competitive binding of FKBP51FK1.

## Discussion

Within the last years, Glmn emerged as a regulator of the E3 ligase Skp1-Cullin-F-box-complex. It is connected to this complex via Cul1 and Rbx1 [3]. Both Glmn knockdown and knockout cells exhibit altered levels of E3 ligase targets [1]. Little is known regarding the regulation of Glomulin itself. FKBPs, especially FKBP52 and FKBP12, were reported to bind to Glmn but comprehensive studies on these interactions are missing. Here, we report that Glmn is able to bind all tested FKBPs, including FKBP51 and FKBP12.6.

The finding that FKBP12 binds Glmn much weaker than the highly homologous 12.6 was highly unexpected. Both small FKBPs are well described for their regulation of ryanodine receptors (RyR), which mediate calcium influx during muscle contraction. Different affinities and selectivity for FKBP12 and FKBP12.6 towards RyR1 and RyR2 have been reported (recently reviewed [13]). FKBP12 and 12.6 differ in 18 amino acids of which three residues (Gln31, Asn32, and Phe59 in FKBP12.6) were sufficient to reverse the selectivity towards RyR2 [28]. Similar swapping experiments may identify the residues underlying the affinity differences of FKBP12 and FKBP12.6.

Surprising differences in Glmn binding were also found for the highly homologous large FKBPs FKBP51 and FKBP52. The higher affinity of FKBP52 has to reside within the FK2 and TPR domains, which seem to form additional contacts with Glmn that are much more productive compared to FKBP51. Future truncation and domain swapping experiments between full-length FKBP51 and FKBP52 will be necessary to further elucidate the role of the FK2 and TPR domains of FKBP51 and FKBP52. Based on crystallographic data, FKBP52 can exhibit two different confirmations: one is similar to FKBP51 and a second conformation where its FK1 domain is rotated by 180° relative to its FK2 domain [19]. This second confirmation might allow additional contacts with Glmn that are unavailable for FKBP51.

In our studies, we identified FKBP52 as a preferred Glmn binding partner over FKBP51. These data predict FKBP52-Glmn complexes to dominate over FKBP51-Glmn complexes unless the latter is highly overexpressed such as suggested in adipocytes [29,30] or skeletal muscle [31,32]. Likewise, our data predict that FKBP12.6-Glmn complexes to dominate over FKBP12-Glmn complexes. The dominant interaction FKBP interaction partner, of course, depends also on the relative expression levels of FKBPs, which is cell-type and context-dependent.

While the strongly reduced activity of the FD67/68DV mutation points to the requirement of an intact FK506-binding pocket and additionally also to a functional role to the PPIase activity, it is possible that these mutations alter the structure of the FKBPs in more global ways that eventually impinge on a reduced capacity to bind to Glmn. In this study, the FK506-binding domain of FKBP51 was used as a model system and functional tracer to allow competitive studies. Due to the high homology within the FKBP family, it seems likely that the FK506-binding site is the major binding site for Glmn for other FKBPs as well. However, it is possible that the FK506 binding site mutations behave differently in the context of the full-length protein. Numerous, high-quality ligands for FKBPs are available [20,33–36], some with substantial FKBP-subtype selectivity [21,37–39]. This suggests that the putative regulatory role of FKBPs on Glmn can be pharmacologically blocked with drug-like molecules.

Our findings may also be relevant for the mechanism of action of FKBP5,1 which recently has raised substantial interests as a risk factor for stress-related diseases, obesity/diabetes, and chronic pain. FKBP51 has been suggested to interact with numerous protein partners but the detailed biochemical mode of actions is so far elusive. To our knowledge, this study provides the first biochemically detailed characterization of a purified protein binding partner of FKBP51.

Collectively, these findings support the hypothesis that FKBPs might represent a new layer of regulation of Glmn and that different FKBPs can differentially regulate the function of Glmn. Taken together, our work defines FKBPs as high-affinity interactions partners of Glmn that may offer a pharmacological perspective to manipulate Glmn-related disorders.

## Supporting information

S1 Fig (A) Structure of TAMRA labelled tracers / quenchers FK[4.3.1]-16g and (B) 2b. (TIF)

S2 Fig Active site titrations of all used FKBPs except for FKBP51FK1 (1–160) FD67/68DV. Assays were performed in duplicates or triplicates with 50 nM TAMRA-FK[4.3.1]-16g tracer. Graphs values of independent dilution series. Active protein concentrations were calculated by c_*AST*_ = ((0.5x c_*Tracer*_ + K_*D*_) / EC_*50*_) x c_*UV*_.
(TIF)

S3 Fig ITC for FKBP51FK1 (1–160) FD67/68DV. To assess the binding-active fraction of the FKBP51FK1 (1–160) sample an ITC experiment was carried out. A 20 μM solution of the protein in 20 mM HEPES pH 8.0, 20 mM NaCl, 5% glycerol and 1% DMSO was placed in the sample cell of a PEAQ-ITC (Malvern) and a 200 μM solution of the ligand FK[4.3.1]-16h in the same buffer was filled into the syringe. After equilibration to 25 °C the ligand was titrated stepwise into the protein solution. The resulting data was analyzed using the provided ITC analysis software and fitted to a one-site binding model yielding the binding enthalpy (ΔH) the K_d_-value and the binding stoichiometry (n).
(TIF)

S1 Table Overview of used competing proteins listing concentrations determined via active site titration, isothermal titration calorimetry and UV absorption as well as the KD values of the tracer FK[4.3.1]-16g to the respective protein. (XLSX)

S1 File Primary data. (XLSX)

S2 File Primary data of the supplemental information. (XLSX)

## References

[1] Tron AE, Arai T, Duda DM, Kuwabara H, Olszewski JL, Fujiwara Y, et al The glomuvenous malformation protein Glomulin binds Rbx1 and regulates cullin RING ligase-mediated turnover of Fbw7. Mol Cell. 2012;46(1):67–78. Epub 2012/03/13. 10.1016/j.molcel.2012.02.005 . 22405651 PMC3336104

[2] Shimizu K, Nihira NT, Inuzuka H, Wei W. Physiological functions of FBW7 in cancer and metabolism. Cell Signal. 2018;46:15–22. Epub 2018/02/24. 10.1016/j.cellsig.2018.02.009 . 29474981 PMC5882551

[3] Duda DM, Olszewski JL, Tron AE, Hammel M, Lambert LJ, Waddell MB, et al Structure of a glomulin-RBX1-CUL1 complex: inhibition of a RING E3 ligase through masking of its E2-binding surface. Mol Cell. 2012;47(3):371–82. Epub 2012/07/04. 10.1016/j.molcel.2012.05.044 . 22748924 PMC3477590

[4] Suzuki S, Suzuki T, Mimuro H, Mizushima T, Sasakawa C. Shigella hijacks the glomulin-cIAPs-inflammasome axis to promote inflammation. EMBO Rep. 2018;19(1):89–101. Epub 2017/12/02. 10.15252/embr.201643841 . 29191979 PMC5757219

[5] Brouillard P, Boon LM, Mulliken JB, Enjolras O, Ghassibe M, Warman ML, et al Mutations in a novel factor, glomulin, are responsible for glomuvenous malformations ("glomangiomas"). Am J Hum Genet. 2002;70(4):866–74. Epub 2002/02/15. 10.1086/339492 . 11845407 PMC379115

[6] Brouillard P, Boon LM, Revencu N, Berg J, Dompmartin A, Dubois J, et al Genotypes and phenotypes of 162 families with a glomulin mutation. Mol Syndromol. 2013;4(4):157–64. Epub 2013/06/27. 10.1159/000348675 . 23801931 PMC3666456

[7] Brouillard P, Ghassibe M, Penington A, Boon LM, Dompmartin A, Temple IK, et al Four common glomulin mutations cause two thirds of glomuvenous malformations ("familial glomangiomas"): evidence for a founder effect. J Med Genet. 2005;42(2):e13 Epub 2005/02/04. 10.1136/jmg.2004.024174 . 15689436 PMC1735996

[8] Suzuki S, Mimuro H, Kim M, Ogawa M, Ashida H, Toyotome T, et al Shigella IpaH7.8 E3 ubiquitin ligase targets glomulin and activates inflammasomes to demolish macrophages. Proc Natl Acad Sci U S A. 2014;111(40):E4254–63. Epub 2014/09/24. 10.1073/pnas.1324021111 . 25246571 PMC4210038

[9] Chambraud B, Radanyi C, Camonis JH, Shazand K, Rajkowski K, Baulieu EE. FAP48, a new protein that forms specific complexes with both immunophilins FKBP59 and FKBP12. Prevention by the immunosuppressant drugs FK506 and rapamycin. J Biol Chem. 1996;271(51):32923–9. Epub 1996/12/20. 10.1074/jbc.271.51.32923 . 8955134

[10] Neye H. Mutation of FKBP associated protein 48 (FAP48) at proline 219 disrupts the interaction with FKBP12 and FKBP52. Regul Pept. 2001;97(2–3):147–52. Epub 2001/02/13. . 11164950 10.1016/s0167-0115(00)00206-8

[11] Taipale M, Tucker G, Peng J, Krykbaeva I, Lin ZY, Larsen B, et al A quantitative chaperone interaction network reveals the architecture of cellular protein homeostasis pathways. Cell. 2014;158(2):434–48. Epub 2014/07/19. 10.1016/j.cell.2014.05.039 . 25036637 PMC4104544

[12] Hahle A, Merz S, Meyners C, Hausch F. The Many Faces of FKBP51. Biomolecules. 2019;9(1). Epub 2019/01/24. 10.3390/biom9010035 . 30669684 PMC6359276

[13] Kolos JM, Voll AM, Bauder M, Hausch F. FKBP Ligands-Where We Are and Where to Go? Front Pharmacol. 2018;9:1425 Epub 2018/12/21. 10.3389/fphar.2018.01425 . 30568592 PMC6290070

[14] Guy NC, Garcia YA, Cox MB. Therapeutic Targeting of the FKBP52 Co-Chaperone in Steroid Hormone Receptor-Regulated Physiology and Disease. Curr Mol Pharmacol. 2015;9(2):109–25. Epub 2015/05/20. 10.2174/1874467208666150519114115 . 25986565

[15] Kozany C, Marz A, Kress C, Hausch F. Fluorescent probes to characterise FK506-binding proteins. Chembiochem. 2009;10(8):1402–10. 10.1002/cbic.200800806 . 19418507

[16] Fabian AK, Marz A, Neimanis S, Biondi RM, Kozany C, Hausch F. InterAKTions with FKBPs—mutational and pharmacological exploration. PLoS One. 2013;8(2):e57508 Epub 2013/03/08. 10.1371/journal.pone.0057508 23469007 PMC3585324

[17] Marz AM, Fabian AK, Kozany C, Bracher A, Hausch F. Large FK506-binding proteins shape the pharmacology of rapamycin. Mol Cell Biol. 2013;33(7):1357–67. Epub 2013/01/30. 10.1128/MCB.00678-12 . 23358420 PMC3624267

[18] Bracher A, Kozany C, Thost AK, Hausch F. Structural characterization of the PPIase domain of FKBP51, a cochaperone of human Hsp90. Acta Crystallogr D Biol Crystallogr. 2011;67(Pt 6):549–59. 10.1107/S0907444911013862 . 21636895

[19] Bracher A, Kozany C, Hahle A, Wild P, Zacharias M, Hausch F. Crystal structures of the free and ligand-bound FK1-FK2 domain segment of FKBP52 reveal a flexible inter-domain hinge. J Mol Biol. 2013;425(22):4134–44. 10.1016/j.jmb.2013.07.041 . 23933011

[20] Pomplun S, Sippel C, Hahle A, Tay D, Shima K, Klages A, et al Chemogenomic Profiling of Human and Microbial FK506-Binding Proteins. J Med Chem. 2018;61(8):3660–73. 10.1021/acs.jmedchem.8b00137 . 29578710

[21] Gaali S, Kirschner A, Cuboni S, Hartmann J, Kozany C, Balsevich G, et al Selective inhibitors of the FK506-binding protein 51 by induced fit. Nat Chem Biol. 2015;11(1):33–7. 10.1038/nchembio.1699 . 25436518

[22] Hartmann J, Wagner KV, Gaali S, Kirschner A, Kozany C, Ruhter G, et al Pharmacological Inhibition of the Psychiatric Risk Factor FKBP51 Has Anxiolytic Properties. J Neurosci. 2015;35(24):9007–16. Epub 2015/06/19. 10.1523/JNEUROSCI.4024-14.2015 . 26085626 PMC6605153

[23] Barent RL, Nair SC, Carr DC, Ruan Y, Rimerman RA, Fulton J, et al Analysis of FKBP51/FKBP52 chimeras and mutants for Hsp90 binding and association with progesterone receptor complexes. Mol Endocrinol. 1998;12(3):342–54. Epub 1998/03/26. 10.1210/mend.12.3.0075 . 9514152

[24] Jagtap PKA, Asami S, Sippel C, Kaila VRI, Hausch F, Sattler M. Selective Inhibitors of FKBP51 Employ Conformational Selection of Dynamic Invisible States. Angew Chem Int Ed Engl. 2019 Epub 2019/05/18. 10.1002/anie.201902994 . 31100184

[25] Wochnik GM, Ruegg J, Abel GA, Schmidt U, Holsboer F, Rein T. FK506-binding proteins 51 and 52 differentially regulate dynein interaction and nuclear translocation of the glucocorticoid receptor in mammalian cells. J Biol Chem. 2005;280(6):4609–16. Epub 2004/12/14. 10.1074/jbc.M407498200 . 15591061

[26] Shim S, Yuan JP, Kim JY, Zeng W, Huang G, Milshteyn A, et al Peptidyl-prolyl isomerase FKBP52 controls chemotropic guidance of neuronal growth cones via regulation of TRPC1 channel opening. Neuron. 2009;64(4):471–83. Epub 2009/12/01. 10.1016/j.neuron.2009.09.025 . 19945390 PMC2786904

[27] Tradler T, Stoller G, Rucknagel KP, Schierhorn A, Rahfeld JU, Fischer G. Comparative mutational analysis of peptidyl prolyl cis/trans isomerases: active sites of Escherichia coli trigger factor and human FKBP12. FEBS Lett. 1997;407(2):184–90. Epub 1997/04/28. 10.1016/s0014-5793(97)00345-1 . 9166896

[28] Xin HB, Rogers K, Qi Y, Kanematsu T, Fleischer S. Three amino acid residues determine selective binding of FK506-binding protein 12.6 to the cardiac ryanodine receptor. J Biol Chem. 1999;274(22):15315–9. Epub 1999/05/21. 10.1074/jbc.274.22.15315 . 10336416

[29] Pereira MJ, Palming J, Svensson MK, Rizell M, Dalenback J, Hammar M, et al FKBP5 expression in human adipose tissue increases following dexamethasone exposure and is associated with insulin resistance. Metabolism. 2014;63(9):1198–208. Epub 2014/07/07. 10.1016/j.metabol.2014.05.015 . 24997500

[30] Sidibeh CO, Pereira MJ, Abalo XM, G JB, Skrtic S, Lundkvist P, et al FKBP5 expression in human adipose tissue: potential role in glucose and lipid metabolism, adipogenesis and type 2 diabetes. Endocrine. 2018;62(1):116–28. Epub 2018/07/23. 10.1007/s12020-018-1674-5 . 30032404 PMC6153563

[31] Su AI, Wiltshire T, Batalov S, Lapp H, Ching KA, Block D, et al A gene atlas of the mouse and human protein-encoding transcriptomes. Proc Natl Acad Sci U S A. 2004;101(16):6062–7. Epub 2004/04/13. 10.1073/pnas.0400782101 . 15075390 PMC395923

[32] Shimoide T, Kawao N, Tamura Y, Morita H, Kaji H. Novel roles of FKBP5 in muscle alteration induced by gravity change in mice. Biochem Biophys Res Commun. 2016;479(3):602–6. Epub 2016/09/30. 10.1016/j.bbrc.2016.09.126 . 27680313

[33] Wang Y, Kirschner A, Fabian AK, Gopalakrishnan R, Kress C, Hoogeland B, et al Increasing the efficiency of ligands for FK506-binding protein 51 by conformational control. J Med Chem. 2013;56(10):3922–35. 10.1021/jm400087k . 23647266

[34] Bischoff M, Sippel C, Bracher A, Hausch F. Stereoselective construction of the 5-hydroxy diazabicyclo[4.3.1]decane-2-one scaffold, a privileged motif for FK506-binding proteins. Org Lett. 2014;16(20):5254–7. 10.1021/ol5023195 . 25286062

[35] Pomplun S, Wang Y, Kirschner A, Kozany C, Bracher A, Hausch F. Rational design and asymmetric synthesis of potent and neurotrophic ligands for FK506-binding proteins (FKBPs). Angew Chem Int Ed Engl. 2015;54(1):345–8. 10.1002/anie.201408776 . 25412894

[36] Gopalakrishnan R, Kozany C, Gaali S, Kress C, Hoogeland B, Bracher A, et al Evaluation of synthetic FK506 analogues as ligands for the FK506-binding proteins 51 and 52. J Med Chem. 2012;55(9):4114–22. 10.1021/jm201746x . 22455444

[37] Gopalakrishnan R, Kozany C, Wang Y, Schneider S, Hoogeland B, Bracher A, et al Exploration of pipecolate sulfonamides as binders of the FK506-binding proteins 51 and 52. J Med Chem. 2012;55(9):4123–31. Epub 2012/03/30. 10.1021/jm201747c . 22455398

[38] Gaali S, Feng X, Hahle A, Sippel C, Bracher A, Hausch F. Rapid, Structure-Based Exploration of Pipecolic Acid Amides as Novel Selective Antagonists of the FK506-Binding Protein 51. J Med Chem. 2016;59(6):2410–22. 10.1021/acs.jmedchem.5b01355 . 26954324

[39] Feng X, Sippel C, Bracher A, Hausch F. Structure-Affinity Relationship Analysis of Selective FKBP51 Ligands. J Med Chem. 2015;58(19):7796–806. 10.1021/acs.jmedchem.5b00785 . 26419422

