# Supplementary figures and images for: FKBP51 and FKBP12.6—Novel and tight interactors of Glomulin
Source: PLoS One. 2019 Sep 6;14(9):e0221926. doi: 10.1371/journal.pone.0221926 (PMC6730887; doi:10.1371/journal.pone.0221926)

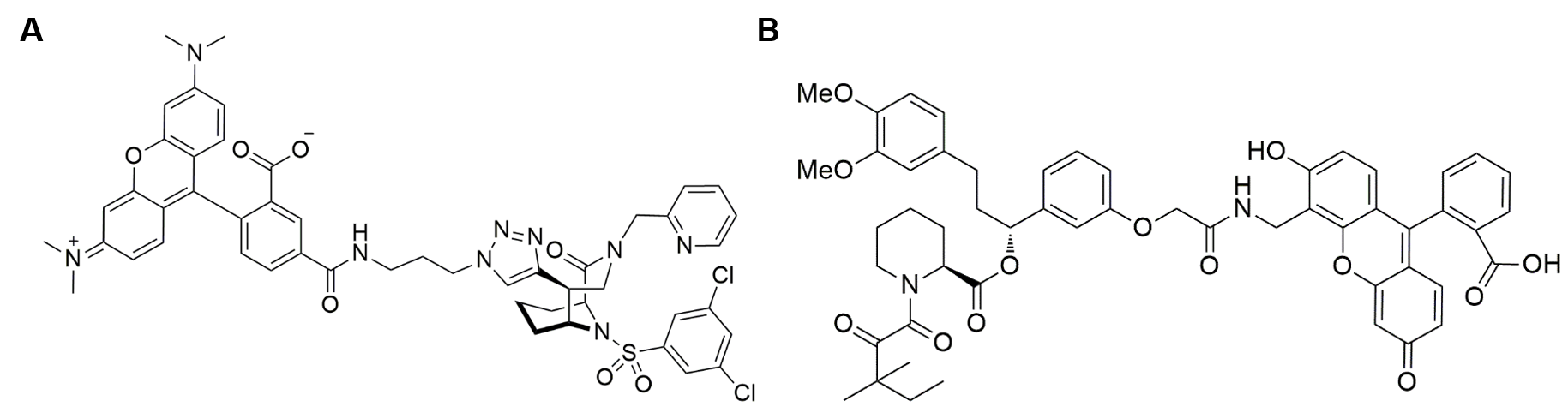

Supplement: S1 Fig — (TIF) [file pone.0221926.s001.tif]
